# *PTCHD1*: Identification and Neurodevelopmental Contributions of an Autism Spectrum Disorder and Intellectual Disability Susceptibility Gene

**DOI:** 10.3390/genes13030527

**Published:** 2022-03-17

**Authors:** Stephen F. Pastore, Sangyoon Y. Ko, Paul W. Frankland, Paul A. Hamel, John B. Vincent

**Affiliations:** 1 Molecular Neuropsychiatry and Development (MiND) Lab, Molecular Brain Science Research Department, Campbell Family Mental Health Research Institute, Centre for Addiction and Mental Health, Toronto, ON M5T 1RS, Canada; stephen.pastore@camh.ca; 2 Institute of Medical Science, University of Toronto, Toronto, ON M5S 1A8, Canada; paul.frankland@sickkids.ca; 3 Program in Neurosciences and Mental Health, The Hospital for Sick Children, Toronto, ON M5G 1X8, Canada; sangyoon.ko@sickkids.ca; 4 Department of Physiology, University of Toronto, Toronto, ON M5S 1A8, Canada; 5 Department of Psychology, University of Toronto, Toronto, ON M5S 3G3, Canada; 6 Department of Laboratory Medicine and Pathobiology, University of Toronto, Toronto, ON M5S 1A8, Canada; paul.hamel@utoronto.ca; 7 Department of Psychiatry, University of Toronto, Toronto, ON M5T 1R8, Canada

**Keywords:** *PTCHD1*, *PTCHD1-AS*, autism spectrum disorders, intellectual disability, neurodevelopment

## Abstract

Over the last one and a half decades, copy number variation and whole-genome sequencing studies have illuminated the considerable genetic heterogeneity that underlies the etiologies of autism spectrum disorder (ASD) and intellectual disability (ID). These investigations support the idea that ASD may result from complex interactions between susceptibility-related genetic variants (single nucleotide variants or copy number variants) and the environment. This review outlines the identification and neurobiological characterization of two such genes located in Xp22.11, Patched domain-containing 1 (*PTCHD1*), and its antisense lncRNA *PTCHD1-AS*. Animal models of *Ptchd1* disruption have recapitulated a subset of clinical symptoms related to ASD as well as to ID. Furthermore, these *Ptchd1* mouse knockout studies implicate the expression of *Ptchd1* in both the thalamic and the hippocampal brain regions as being crucial for proper neurodevelopment and cognitive function. Altered kynurenine metabolic signalling has been postulated as a disease mechanism in one of these animal studies. Additionally, ASD patient-derived induced pluripotent stem cells (iPSCs) carrying a copy number loss impacting the antisense non-coding RNA *PTCHD1-AS* have been used to generate 2D neuronal cultures. While copy number loss of *PTCHD1-AS* does not affect the transcription of *PTCHD1*, the neurons exhibit diminished miniature excitatory postsynaptic current frequency, supporting its role in ASD etiology. A more thorough understanding of risk factor genes, such as *PTCHD1* and *PTCHD1-AS*, will help to clarify the intricate genetic and biological mechanisms that underlie ASD and ID, providing a foundation for meaningful therapeutic interventions to enhance the quality of life of individuals who experience these conditions.

## 1. Introduction

Autism spectrum disorders (ASD) have an estimated global prevalence of 1–2% [1]. The role of genetics in ASD is strongly supported by twin studies, which collectively suggest heritability rates as high as 80% [2]. Furthermore, the frequency of ASD in males is over three-fold higher than in females. One suggested cause for at least a portion of this skewed ratio could be genetic determinants for ASD on the X chromosome [3]. With the advent of whole genome microarrays and next-generation sequencing (NGS) technologies, studies began to investigate ASD on a genome-wide level. These efforts rapidly confirmed that the etiology of ASD is multigenic and highly heterogeneous. Furthermore, many of the putative ASD risk loci display a high degree of pleiotropy. More than 70% of individuals with ASD have concurrent developmental, psychiatric, or behavioural conditions, including intellectual disability (ID) (45%), attention deficit hyperactivity disorder (ADHD) (28–44%), motor abnormalities (as high as 79%), sleep disorders (50–80%), epilepsy (8–30%), hyper-aggression (as high as 68%), and anxiety (42–56%) [1].

The current understanding is that both ASD and ID may share many of the same susceptibility genes and variants, as well as complex interactions between genetics and the environment [4]. This review will describe the discovery and subsequent characterization of the ASD and ID susceptibility gene Patched domain-containing 1 (*PTCHD1*) (MIM:300828), as well as the antisense lncRNA *PTCHD1-AS*, which are both located on the short arm of the X chromosome. We will focus particularly on *PTCHD1* regulation in the brain and its corresponding neurobiological function, with additional emphasis placed on *Ptchd1* mouse models of atypical behaviour and neurodevelopment.

### 1.1. Identification of PTCHD1 and PTCHD1-AS as ASD and ID Susceptibility Genes

In 2008, a study of 427 ASD probands identified 277 unique CNVs across the genome that were absent in neurotypical controls. One of the elucidated CNVs consisted of a 167 Kb deletion in Xp22.11 that, in congruence with canonical X-linked patterns of inheritance, was transmitted from an unaffected carrier mother to both a male ASD proband and his developmentally delayed dizygotic twin brother. This microdeletion encompassed the first exon of *PTCHD1*, resulting in a null allele, as well as exons of the *PTCHD1-AS* ncRNA [5].

### 1.2. Rare PTCHD1 and PTCHD1-AS Variants

**Figure 1:**
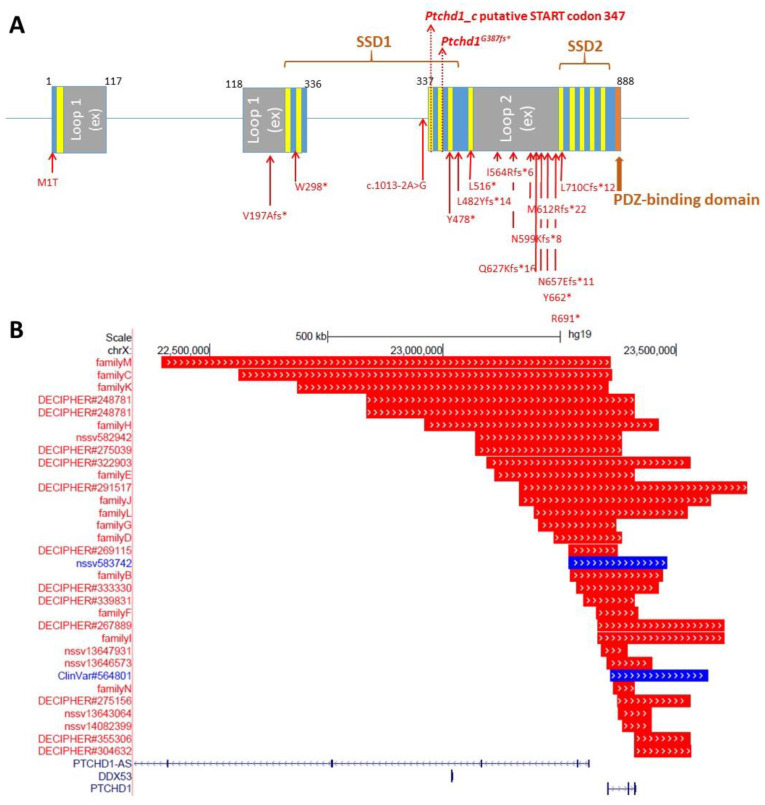
(**A**) Topographic representation of *PTCHD1*/*Ptchd1* genomic structure, with protein domain structure superimposed, and the locations of all known clinically reported loss of function mutations. The positions of the mouse knockout as well as the putative START for the *PTCHD1_c*/*Ptchd1_c* alternatively spliced transcript are marked. The predicted transmembrane domains are indicated in yellow. External loops 1 and 2 and putative sterol-sensing domains (SSD) 1 and 2 are predicted through structural comparison with the homologous protein NPC1. The 10 loss-of-function coding mutations shown here are (from left to right): NM_173495.2:c.2T > C; p.Met1Thr (rs1064796945; ClinVar#424379); NM_173495.2:c.1433dupA; p.Tyr478* (ClinVar#423632); NM_173495.2:c.1444delC; p.Leu482Tyrfs*14 (rs878854361; Chaudhry et al., 2015 [6]; Wright et al., 2014 [24]; DECIPHER:#263331; ClinVar#209087); NM_173495.2:c.1547T > A; p.Leu516* (ClinVar#280396); NM_173495.2:c.1689_1690delTA; p.Ile564Argfs*6 (DECIPHER:#318071; Grozeva et al., 2015 [25]); NM_173495.2:c.1796insA;p.Asn599Lysfs*8 (Chaudhry et al., 2015 [6]; ClinVar#209086); NM_173495.2:c.1835_1839delTGTTGinsGAA; p.Met612Argfs*22 (ClinVar#208739); NM_173495.2:c.1969_1972delAACA; p.Asn657Glufs*11 (ClinVar#372479); NM_173495.2: c.1985_1986del; p.Tyr662* (Z. Stark, Victoria Clinical Genetics Service, *personal communication*); NM_173495.2:c.2071C > T; p.Arg691* (DECIPHER: #259242); NM_173495.2:c.2128delC; p.Leu710Cysfs*12 (Chaudhry et al., 2014 [6]; ClinVar#209085). (**B**) Pathogenic copy number variants impacting *PTCHD1*, using UCSC Genome Browser. Red indicates loss, and blue indicates gain. Families B to N were reported in Chaudhry et al., 2015 [6]; others have been listed by the DECIPHER project (www.deciphergenomics.org, accessed on 1 August 2021), or by ClinVar (www.ncbi.nlm.nih.gov/clinvar, accessed on 1 August 2021) [20], or in Zarrei et al. [15].

A multitude of subsequent genome-wide investigations have corroborated the initial implication by Marshall et al. that *PTCHD1* and *PTCHD1-AS* are susceptibility genes for the development of ASD and ID [5]. Since the initial discovery, almost 70 additional rare genomic variants have been identified (Figure 1; Supplementary Table S1; www.PTCHD1-base.com, accessed on 1 August 2021). These consisted of both microdeletions, which ranged from approximately 46 Kbp to 1.2 Mbp and encompassed portions of *PTCHD1* and/or *PTCHD1-AS* [6,7,8,9,10,11,12,13,14,15], as well as single-nucleotide variants (SNVs) within the coding sequence of *PTCHD1*. Of the SNVs that have been identified, 18 were inherited missense variants, four were truncating variants (one of which was *de novo*), and one was an inherited nonsense mutation [6,8,11,16,17,18,19]. In total, 44 inherited and two *de novo* deletion CNVs, as well as 23 SNVs, have been reported in 69 unrelated probands with neurodevelopmental disorders, 67 of which are male. Notably, these deleterious alleles were almost exclusively maternally transmitted to males affected with an array of cognitive impairments, including ASD, ID, and developmental delays. Entries for additional rare CNVs affecting *PTCHD1* and/or *PTCHD1-AS* exist in clinical genomic databases, although clinical information is sparse or absent for these individuals [8,20,21].

**Figure 2:**
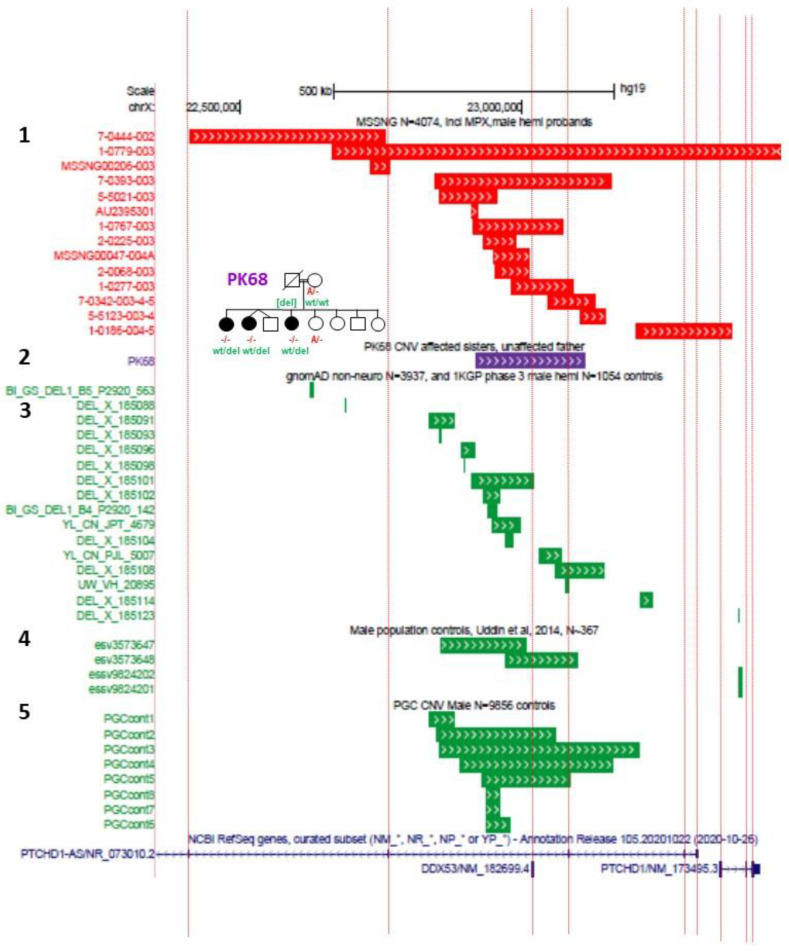
Male loss CNVs across ASD and control populations: 1. Loss CNVs (high-quality rare calls) in male affected individuals from the MSSNG ASD whole genome sequencing project (N = 4074); 2. the position of the loss CNV reported in three intellectual disability females from Pakistani family PK68 (Harripaul et al., 2018) [10], in which the CNV is not inherited from their mother but from deceased unaffected father. Genotypes for loss-of-function mutation in *SLAIN1* (identified through homozygosity-by-descent mapping and whole exome sequencing) are shown in red, and *PTCHD1-AS* loss CNV in green. SLAIN1 is associated with axonal growth during neuronal development (MIM 610491) [26]; 3. gnomAD (https://gnomad.broadinstitute.org/, accessed on 1 December 2021) non-neuro subset controls, N = 3937 males, and 1000 Genome Project phase 3 males, N = 1054); 4. population male controls, N~367 (Uddin et al., 2014) [27]; 5. PGC controls from SCZ study, N = 9856 (Marshall et al., 2017) [28]; Chisquare calculation with Yates’s correction (2-tailed, using www.graphpad.com, accessed on 1 November 2021) for a) MSSNG cases *versus* all control groups and b) for CNVs disrupting *PTCHD1-AS* exon 3 (but not *PTCHD1*) and for all CNVs, except those disrupting *PTCHD1*, are non-significant. (a) Affected: 3 CNV: 4071 no CNV; unaffected: 6 CNV: 15,208 no CNV, *p* = 0.6246; (b) affected: 12 CNV: 4062 no CNV; unaffected 27 CNV: 15,187 no CNV, *p* = 0.2001).

Where both the clinical and the genomic data from immediate family members are available, deletion CNVs that encompassed a portion of *PTCHD1-AS*, but that did not disrupt *PTCHD1*, showed incomplete segregation with the neurodevelopmental phenotype 53% of the time (10 of 19 families) [6,7,11,12,14], suggesting a more complex or subtle contribution of *PTCHD1-AS* to ASD and ID. Conversely, missense variants affecting *PTCHD1* demonstrated a much higher penetrance, segregating with disease at a frequency of 88% (seven of eight families) [11]. In addition, the two microdeletions affecting *PTCHD1-AS* that also ablated a portion of *PTCHD1* (GOLD540 and Family B) both segregated with disease [6,7,11,14]. In 2018, we reported on SNVs and CNVs in consanguineous populations, including a Pakistani family in which a large heterozygous upstream deletion was identified in three affected female siblings. However, the loss CNV was determined to be inherited from their unaffected father, and an alternative candidate mutation was identified in an autosomal gene, *SLAIN1*, within a region of homozygosity-by-descent (Figure 2) [10]. Likewise, it is also important to note that loss CNVs in the *PTCHD1* upstream region, including the exons of *PTCHD1-AS* and *DDX53*, are present in control population males (gnomAD) [22], and in males in both case and control large-scale research study cohorts, such as PGC schizophrenia (Figure 2) [23]. In contrast, loss CNVs spanning the coding exons of *PTCHD1* have, to date, not been found in male controls. We attempted to analyse the numbers of upstream/*PTCHD1-AS* loss CNVs reported in ASD males compared to those reported for male population controls. Although the methodologies for generating data and calling the CNVs from these different sources were non-identical and thus there were risks about drawing conclusions, our analysis suggests that any trends towards increased loss CNV numbers in ASD are non-significant (Figure 2). Collectively, these data indicate a more robust association between *PTCHD1* and aberrant neurodevelopment than appears to be evident for *PTCHD1-AS*. Moreover, it is worth pointing out several notable observations here: (1) the vast majority of truncating coding mutations in *PTCHD1* are located within exon 3, and (2) loss CNVs impacting *PTCHD1* involve either exon 1 (and the upstream region), exons 1–3, exons 2 and 3, or exon 3 alone, and no CNVs remove only exon 2 (Figure 1).

### 1.3. Common PTCHD1 Variants

To supplement the database of rare *PTCHD1/PTCHD1-AS* mutations, Torrico et al. sought to employ tag SNP genotyping to identify common *PTCHD1* variants that may be associated with ASD and ID. A tag SNP is representative of a group of SNPs that is inherited together due to linkage disequilibrium, and therefore, this approach can identify *PTCHD1/PTCHD1-AS* haplotypes that are associated with ASD. The authors genotyped 595 individuals with ASD and concluded that the haplotype inferred by the tag SNP rs7052177, which is located within intron 1, displayed a significant association with ASD and ID. The rs7052177 SNP is located within a putative binding site for the transcription factors STAT3, STAT5A, and STAT5B, which are all predicted to bind preferentially to the rs7052177T allele, implying that this common variant may have a functional influence on the regulation of *PTCHD1*. This study also identified several short duplications and deletions, and one simple tandem repeat, within the *PTCHD1* promoter regions in both the control and the ASD cohorts. Most notable among these findings was a 27 bp duplication within the promoter region that was present in three individuals with ASD; subsequent luciferase analyses determined that this duplication attenuated reporter gene activity by 26% in vitro. In addition, a GCC trinucleotide repeat was detected 80 bp upstream of the *PTCHD1* transcription start site (TSS); a follow-up case-control association study confirmed that the longest allele of this microsatellite (14 repeats) was associated with ASD [19].

### 1.4. Clinical Presentation of PTCHD1 and PTCHD1-AS Variants

The investigation by Chaudhry et al. also reported on clinical assessments of 23 patients with either loss-of-function SNVs or loss CNVs disrupting the *PTCHD1* gene in order to understand better the genotype–phenotype relationship and to look for commonalities that may be useful for clinical diagnosis [6]. In this regard, no significant growth abnormalities were reported in the majority of these cases, although two patients (9%) had an early failure to thrive. Four subjects (18%) had relative or absolute macrocephaly and three (13%) had relative or absolute microcephaly. These authors also report that most participants were non-dysmorphic, but others presented with combinations of minor facial dysmorphic features, including a long face, a prominent forehead, puffy eyelids, a narrow face with a small chin, pronounced upper central incisors, a depressed and narrowed nasal bridge with a broad nasal tip and anteverted nostrils, and a thin upper lip. The most consistently observed facial dysmorphic feature in this study was an open-mouth posture and secondary orofacial hypotonia, which was observed in 11 of the 23 individuals (48%). Cognitively, 18 cases (78%) displayed a global developmental delay in early childhood. In addition, nine patients (39%) had formal diagnoses of ID. Eight subjects (35%) had a diagnosis of ASD and another two demonstrated ASD-like features but did not have formal diagnoses. Behavioural problems, such as mood disorders and hyper-aggression, were also reported in four individuals (18%). Neurologically, six participants (26%) presented with generalized hypotonia, and two others (9%) were reported to have mild peripheral hypertonia. In addition, five individuals (22%) had poor balance and gait abnormalities. Lastly, eight patients (35%) possessed mild vision problems, including strabismus, jerky oculomotor movements, cataracts, astigmatism, and myopia [6].

## 2. Expression Profiling of *PTCHD1* and *PTCHD1-AS*

### 2.1. Gene Structure and Alternative Splicing

*PTCHD1* spans almost 70 Kb on the forward strand of the short arm of the X chromosome and has two annotated protein-coding transcripts. The larger transcript (NM_173495.3) is almost 13 Kb and contains three exons that encode for 888 amino acids. Alternative splicing is evident, and a brain-specific transcript excluding the second exon has been identified (GenBank ID KR270726) and contains a 542 amino acid-encoding open reading frame (ORF), which includes the majority of the transmembrane domains and the C-terminal PDZ-binding domain, although it deletes entirely the first luminal domain and a portion of the sterol-sensing domain-like module present in the 888 amino acid ORF. *PTCHD1-AS* (NR_073010.2) is encoded on the reverse strand and is divergently transcribed with respect to *PTCHD1*.

*PTCHD1-AS* appears to be spliced into at least three known splice variants (*AS1*, *AS2* and *AS3*), with at least 10 distinct exons having been identified. Several of these exons are conserved across all *PTCHD1-AS* splice variants. Interestingly, the third exon of the splice variants *PTCHD1-AS2* and *PTCHD1-AS3* (which is also the putative second exon in *PTCHD1-AS1*) is expressed in the antisense transcripts at syntenic loci from both mice and rats, indicating some degree of evolutionary conservation [11]. Two putative TSSs have been identified for *PTCHD1-AS*, with each mapping to the first exon of either *PTCHD1-AS2* or *PTCHD1-AS3* [29]. The TSSs for *PTCHD1-AS3* and *PTCHD1* are separated by approximately 40 Kb, suggesting that expression of these genes may be governed by a bi-directional promoter. ChIP-seq. data from human embryonic stem cell-derived neurons appear to support this theory as this region was found to be enriched in histone markers corresponding to both promoters (H3K4me3) and enhancers (H3K27ac and H3K4me1). Notably, the putative TSS that allegedly regulates *PTCHD1-AS2* is only associated with enhancer histone markers [30].

### 2.2. Spatial and Temporal Expression during Neurodevelopment

Comprehensive transcriptional analyses using RT-PCR by Noor et al. revealed that the *PTCHD1* full-length (isoform a) transcript exhibits varying levels of expression in the human brain, as well as in numerous peripheral tissues, with the highest levels of transcription being evident in the cerebellum. A subsequent northern blot analysis revealed that, in addition to the cerebellum, the *PTCHD1* transcript was also detected in all of the four major lobes of the brain. The authors then employed qRT-PCR to compare the expression of *PTCHD1* in multiple brain subregions, confirming the highest relative levels of transcription in both the cerebellum and the pituitary gland. Noor et al. also used RT-PCR to characterize the expression of *PTCHD1-AS1* and *PTCHD1-AS2* and reported detectable levels of both transcripts in the cerebellum, parietal and occipital lobes, spinal cord, and fibroblasts [11].

Studies employing RNA in situ hybridization and fluorescent in situ hybridization (FISH) characterized the spatial expression profile of *Ptchd1* in mice during embryogenesis and postnatally. Noor et al. first reported extensive expression of the *Ptchd1* transcript throughout the developing brain during embryogenesis (E9 and E14) through RNA in situ hybridization [11]. Furthermore, at birth (P0), *Ptchd1* mRNA appears to be primarily restricted to the thalamic reticular nucleus (TRN). Beginning in adolescence (P15), however, *Ptchd1* expression becomes detectable in the cortex, striatum, cerebellum, and dentate gyrus of the hippocampus [31]. Consistent with these reports, Tora et al. confirmed high relative levels of *Ptchd1* expression in both the TRN and the cerebellum at P12, as well as in the dentate gyrus at P21. Interestingly, *Ptchd1* transcription in the dentate gyrus at P21 appears to be almost entirely restricted to dentate granule cells, with very low expression observed in the pyramidal cells in other hippocampal subfields (CA1–3). Furthermore, considerable *Ptchd1* expression was observed in the dentate gyrus of adult (P60) mice [32]. In addition to the dentate gyrus and TRN, *Ptchd1* exhibits strong expression in the anterodorsal subdivision (AD) of the thalamus in adult mice [13].

Tora et al. subsequently used qRT-PCR in order to quantitatively profile *Ptchd1* expression throughout early postnatal development and into adulthood (P5–P60) in these relevant brain subregions. These analyses revealed that *Ptchd1* expression declined in the thalamus after P5 and, conversely, increased in the cerebellum after P15; no postnatal changes in expression were observed in the cortex, hippocampus, or striatum [32]. Ung et al. also report variable embryonic and postnatal expression of *Ptchd1* between numerous additional brain subregions, such as the midbrain, pons, medulla, olfactory bulb, and hypothalamus, as well as dynamic expression within these subregions between the embryonic (E13–E18) and postnatal (P7 and P35) ages that were assessed [33].

### 2.3. Neuronal-Activity-Dependent Transcription

A comprehensive genome-wide study provided evidence that the transcription of *Ptchd1* can be upregulated in response to neuronal activity. In this study, Kim et al. depolarized primary mouse cortical neurons with potassium chloride for six hours and subsequently performed RNA sequencing to identify activity-dependent genes. Interestingly, *Ptchd1* exhibited a three-fold increase in expression in response to this stimulus [34]. The activity-dependent expression of *PTCHD1* was subsequently corroborated by Ross et al., who reported an almost two-fold depolarization-induced increase in *PTCHD1* expression in human induced pluripotent stem cell (iPSC)-derived cortical neurons. Notably, the expression of *PTCHD1-AS* was unchanged in human iPSC-derived cortical neurons in response to neuronal depolarization [35]. These data imply that the neuronal function of PTCHD1, but not *PTCHD1-AS*, may be related to strengthening intracellular signalling cascades within the postsynaptic density following long-term potentiation.

### 2.4. Putative Synapse-Localized Translation

Ascano et al. employed photoactivatable ribonucleoside-enhanced crosslinking and immunoprecipitation (PAR-CLIP), followed by RNA sequencing, in order to detect global targets of the RNA-binding protein Fragile X Mental Retardation Protein 1 (FMRP) in vitro. Interestingly, the authors report a modest interaction between FMRP and the *PTCHD1* transcript, with a putative 22 bp binding element identified in the 3′ untranslated region [36]. This putative interaction between *PTCHD1* mRNA and FMRP suggests an additional layer of *PTCHD1* regulation in neurons, in which local synaptic translation is mediated in response to the group 1 metabotropic glutamate receptor (mGluR)-activation of FMRP.

### 2.5. Functional Characterization of PTCHD1

Studying the function of the PTCHD1 protein will enable a better understanding of the biological pathways leading to ASD and ID, as well as providing the possibility of more accurate clinical prediction for *PTCHD1* sequence variants, and in particular for missense variants, and the first steps towards considering therapeutic strategies.

**Figure 3:**
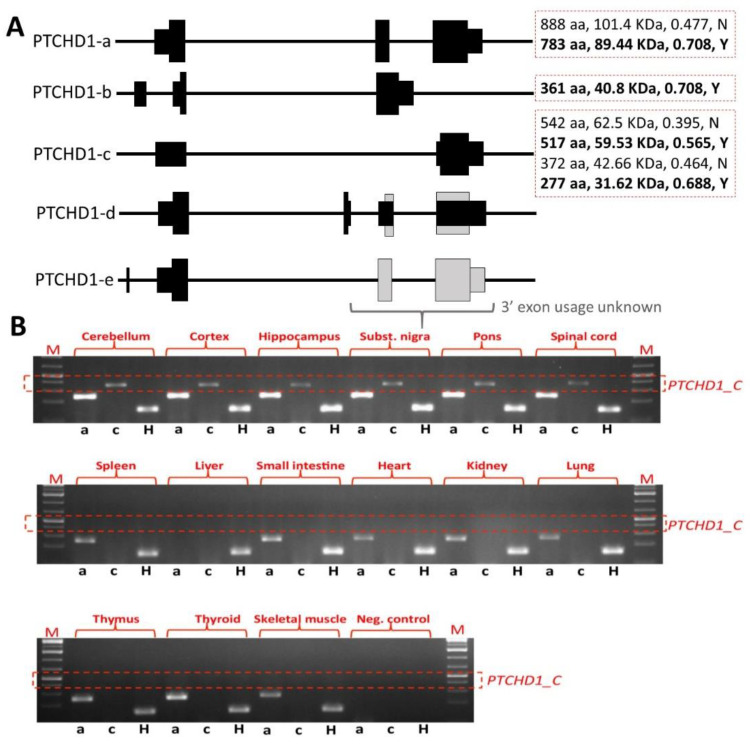
Alternative splicing cartoon and tissue specificity of *PTCHD1*. (**A**) Alternatively spliced transcripts were identified through genes, mRNAs and expressed sequence tags (ESTs) from UCSC Genome Browser (https://genome.ucsc.edu, accessed 1 February 2022) and through our own unpublished transcriptional studies (deposited in GenBank by Vincent, Mittal, and Degagne, 2006). Four main splice variants are shown, designated a-d. *PTCHD1-a* is the canonical three-exon form, from gene sequence NM_173495.3 (supported by many mRNAs and ESTs) and encodes an 888 amino acid (aa) open reading frame (ORF). Coordinates (hg19) for the exons are: 23,352,985–23,353,343; 23,397,708–23,398, 368; and 23,410,648–23,414,918. *PTCHD1-b*, a three-exon form, is reported as a UCSC Gene but comes from a single cDNA sequence, from teratocarcinoma, BC062344 (from cDNA clone IMAGE#6579014). Coordinates (hg19): 23,352,132–23,352,412; 23,353,181–23,353,343; and 23,397,708–23,399,551. This cDNA utilizes a non-canonical splice acceptor site and was not supported by any additional human or non-human mRNA or EST sequences in the UCSC browser (accessed on 1 February 2022). *PTCHD1-c* was identified from brain tissue cDNAs (KR270726 and KP940348) and skips the second exon. This isoform was supported by RT-PCR studies in human and mouse, but, unlike *PTCHD1-a*, appears to be brain specific. Coordinates (hg19): 23,352,293–23,353,343; and 23,410,648–23,414,918. *PTCHD1-d* identifies a four-exon splice variant, from a single cDNA from whole brain (KJ535090). Coordinates (hg19): 23,352,993–23,353,343; 23,394,845–23,395,008; 23,397,708–23,398,368; and 23,410,648–23,412,298. The mRNA encodes a predicted 5′ ORF corresponding to the N-terminal 124 amino acids of the full-length *PTCHD1-a* 888 aa ORF. However, a larger downstream ORF is also plausible, corresponding to 665 aa from the C-terminal portion of *PTCHD1-a* (indicated by grey boxes). *PTCHD1-e* identifies an upstream exon (further upstream than *PTCHD1-b*). It utilizes the canonical exon 1 from *PTCHD1-a*; however downstream exon usage could not be determined (Vincent et al., unpublished). Predicted open reading frames are shown, indicating number of amino acids (aa), predicted molecular size (using https://www.bioinformatics.org/sms/prot_mw.html, accessed on 1 February 2022, and strength of translation start site, using NetStart 1.0 (https://services.healthtech.dtu.dk/service.php?NetStart-1.0, accessed on 1 February 2022), indicating score and whether predicted (in bold) or not. (**B**) Transcription of human *PTCHD1-a* and *PTCHD1-c* in brain (upper gel) and non-CNS (middle and lower gels) tissues by RT-PCR, using a multi-tissue panel (Origene Technologies, Rockville, MD) of first-strand cDNAs (Vincent et al., unpublished data). *PTCHD1-a* was expressed in brain and non-CNS tissues, whereas *PTCHD1_C* was only detected in brain. cDNA was synthesized using reverse transcription of 1µg of RNA using Superscript III^TM^ Reverse Transcriptase (Invitrogen, Carlsbad, CA, USA) and random hexamers (100 ng) in a 20 µL reaction volume according to manufacturer’s guidelines. *PTCHD1-a* was amplified using primers F: ccgcgtatcagaacgttacc, R: cccatataatccatgacctagca; *PTCHD1-c* was amplified using primers F: cttgaggacgtgtttct, R: catataatccatgacctttaag; housekeeping gene (H) *HPRT* was amplified using F: tggtcaggcagtataatccaaa, R: tcaagggcatatcctacaacaa. The negative control shown was a ‘no template control’.

Preliminary in silico analyses indicate that PTCHD1 is predicted to be a transmembrane protein that encodes the 12 transmembrane helices that form two modules, similar to the sterol-sensing domains in NPC-1, as well as a C-terminal PDZ-binding motif encoded by the final four amino acids (Ile-Thr-Thr-Val, or ITTV; see Figure 3). In order to acquire an initial understanding of the function of PTCHD1, Noor et al. first sought to study its subcellular localization. The authors reported that a C-terminal PTCHD1-GFP fusion protein primarily exhibited localization to the plasma membrane in both COS7 and SK-N-SH (human neuroblastoma) cells in vitro [11]. Ung et al. characterized the subcellular localization of Ptchd1 in neuronal cells by fusing GFP to either its N-terminus (GFP-Ptchd1) or C-terminus (Ptchd1-GFP) and then transiently expressing these fusion proteins in primary hippocampal neuronal cultures ex vivo. These analyses indicated that this exogenously expressed Ptchd1-GFP exhibits a distinct pattern of membrane localization within dendritic spines, which was further corroborated by co-labelling experiments with the post-synaptic density protein Psd95. In addition, the authors report that a portion of the intracellular C-terminal tail encoded by amino acids 850–873 appears to be essential for dendritic and synaptic targeting. Interestingly, GFP-Ptchd1 displayed ubiquitous fluorescence throughout the neuronal cells, suggesting that post-translational processing of the N-terminus of Ptchd1 is necessary for appropriate subcellular localization [33].

Although many *PTCHD1* missense variants have been reported (e.g., Noor et al.) or listed in ClinVar, and even though many of these are exceedingly rare variants, it is not currently possible to assign consequential pathogenicity to them. In order to enable more accurate clinical diagnoses for *PTCHD1* missense variants, it is important to establish empirical methods (rather than in silico predictions) that can identify a link between the variants and PTCHD1 function and thus etiopathological role. With this in mind, Halewa et al. sought to determine the likely pathogenicity of a number of reported *PTCHD1* missense variants by assessing both their protein stability and their plasma membrane localization in vitro. In order to evaluate protein stability, wildtype and missense PTCHD1-GFP fusion protein constructs were transiently overexpressed in HEK293T cells. Subsequent western blotting revealed substantial decreases in the protein expression of the missense variants Pro32Arg, Pro32Leu, Lys181Thr, Tyr213Cys, Gly300Arg, and Ala310Pro, likely indicating conformational instability and resultant degradation. Consistent with these findings, transient overexpression in both HEK293T and Neuro-2a cells, followed by immunostaining, demonstrated that the same six missense variants displayed weak localization at the plasma membrane. Interestingly, these mutations are all clustered within either the extracellular loop (Lys181Thr and Tyr213Cys) or one of the transmembrane domains (Pro32Arg, Pro32Leu, Gly300Arg, and Ala310Pro), implying that mutations in these locations are particularly deleterious and are likely to be retained by the endoplasmic reticulum and targeted for subsequent proteasomal degradation [16]. In a similar vein, Xie et al. conducted cycloheximide chase assays following the transient overexpression of GFP-PTCHD1 in HEK293T cells and concluded that wild type PTCHD1 exhibits a half-life of greater than 12 h. This assay also inferred similar levels of protein stability for the Pro75Gln, Lys181Thr, Gly303Arg, and Tyr802Cys missense variants as well as the PDZ-binding domain deleting mutation I885*, in comparison with the marked instability of Pro32Arg and Phe549Cys. In addition, Xie et al. stably expressed GFP-PTCHD1 missense variants in HEK293T cells, and despite modest 5–10% attenuations in the mRNA expression of the Pro32Arg, Gly303Arg, and Phe549Cys transcripts, 40–80% decreases in their basal protein expression were observed, which shows further consistency with significant increased instability. However, in contrast with Halewa et al., Xie et al. were able to detect Lys181Thr, both basally and following transient overexpression [37]. A summary of the functional studies through missense variants is provided in Table 1.

**Table 1:** Summary of functional analysis of missense PTCHD1 variants from Xie et al. [37] and Halewa et al. [16].

		Xie et al.					Halewa et al.			
PTCHD1 Variant	Minor Allele Frequency (gnomAD)/No. Hemizygotes	Source (ID)	Inheritance	Post-Translational Defect	Protein Stability Reduced	Protein Localization Affected	Source	Inheritance	Protein Stability Reduced	Impaired Plasma Membrane Localization
P32R	0/0	DECIPHER: 284363	Mat	+	+	-	Lille	Mat	+	+
P32L	0/0						Lyon	Mat	+	+
S51N	0/0						Torrico	NR	-	-
L73F	5.36 × 10^−^^5^/2		Mat			-	Noor	Mat	-	-
P75Q	0/0	MSSNG: AU3794302	NR	+	-	-				
I173V	3.24 × 10^−4^/14						Noor	Mat	-	-
K181T	0/0	Clinvar	NR	+	+	-	Karaca, 2015 [17]	Mat	+	+
V195I	0/0						Noor	Mat	-	-
Y213C	0/0						Paris/ Strasbourg	Mat	+	+
G300R	0/0						Lille	Mat	+	+
G303R	0/0	ClinVar ID 417957	NR	+	-	-				
A310P	0/0						Paris	De novo	+	+
H359R	0/0						Noor	Mat	-	-
A470D	0/0						Noor	Mat	-	-
E479G	0/0						Noor	Mat	-	-
F549C	0/0	Ptchd1-base.com	NR	+	+	-				
Q884*	0/0	No subject	NA			-				

NR = not reported; NA = not applicable.

PTCHD1 shares homology (21.17% amino acid identity, as determined by multiple sequence alignment, using Clustal Omega, www.ebi.ac.uk, accessed on 1 February 2022) with Niemann-Pick disease type C1 (NPC1), which is a multi-pass transmembrane protein that possesses 19 residues for N-linked glycosylation [38]. Given this apparent structural homology, Xie et al. hypothesized that disease-associated missense variants of PTCHD1 may lead to aberrant protein processing by the endoplasmic reticulum and Golgi apparatus. Transient overexpression of GFP-PTCHD1 in HEK293T cells and subsequent treatment with either Endoglycosidase H or the amidase PNGase F, followed by SDS-PAGE, revealed that PTCHD1 consistently undergoes N-linked glycosylation [37]. This finding is in agreement with in silico analyses, which predict 10 sites for N-linked glycosylation in PTCHD1 [39]. Furthermore, in addition to N-linked glycosylation with mature complex oligosaccharides, PTCHD1 transiently exists in two putative intermediate states of N-linked glycosylation with immature mannose-rich oligosaccharides. Xie et al. next evaluated N-linked glycan processing in several PTCHD1 missense variants and reported that Pro75Gln, Lys181Thr, Tyr802Cys, and Ile884* all demonstrate the presence of N-linked glycosylation with complex oligosaccharides, at varying levels. In contrast, Pro32Arg, Gly303Arg, and Phe549Cys all fail to achieve N-linked glycosylation with mature glycans, although the two alleged intermediate N-linked glycosylation states were observed for Gly303Arg and Phe549Cys. Interestingly, only one of these supposed intermediate states was observed for Pro32Arg, indicating that this missense variant is particularly resistant to N-linked glycosylation. Regardless, defects in N-linked glycan processing of PTCHD1 missense variants did not appear to cause protein retention in either the endoplasmic reticulum or the Golgi apparatus, suggesting the possibility that PTCHD1 is trafficked via an unconventional secretory pathway [40].

In addition to NPC1, PTCHD1 exhibits sequence homology with the Patched domain-containing proteins Patched-1 (PTCH1; 21.62% identity) and Patched-2 (PTCH2; 20.65% identity), both of which are transmembrane receptors that negatively regulate the Hedgehog (Hh) signalling pathway. Binding of the Hh ligand to PTCH1 inhibits its repression of the G protein-coupled receptor, Smoothened (SMO), and initiates a signalling cascade that ultimately leads to activation of the GLI family of transcription factors [41]. Based on its sequence similarity, it was hypothesized that PTCHD1 may also behave similarly to Ptch1 in the Hh-signalling pathway. To explore this, Noor et al. transiently overexpressed a reporter vector with multimerized GLI transcription factor binding elements and either PTCH1, PTCH2, or PTCHD1 in the Hh-responsive cell line 10T1/2 and reported that PTCH1, PTCH2, and PTCHD1 all inhibit GLI-dependent transcription in vitro [11]. However, Ung et al. showed that PTCHD1 was incapable of rescuing the canonical sonic hedgehog (SHH) pathway in cells depleted of PTCH1, which would suggest PTCHD1 to function in a separate pathway (Ung et al.). Hh-signalling has been implicated in early postnatal granule cell proliferation in both the cerebellum [42] and the dentate gyrus [43]. Moreover, the overproliferation of granule cell precursors leading to medulloblastoma has been reported in mice with homozygous or heterozygous deletions in *Ptch1*, the mouse ortholog of *PTCH1* [44]. In this regard, Tora et al. sought to assess the consequences of *Ptchd1* ablation on these populations of granule neuron precursors using the thymidine analogue BrdU. Unexpectedly, the authors indicate that the granule cell precursors in both the cerebellum and the dentate gyrus do not exhibit increased proliferation in *Ptchd1*^Δ*2/Y*^ mice, which contain a deletion of exon 2, during early postnatal development (P4), nor do the cells in the adult dentate gyrus. Immunocytochemistry subsequently revealed that Shh-binding to ectopically expressed Ptchd1 was undetectable in COS7 cells or mouse embryonic fibroblasts in vitro [32]. Supporting these findings, Ung et al. report that exogenous Ptchd1 does not repress GLI-dependent transcription in mouse embryonic fibroblasts derived from *Ptch1^−/−^* mice [33]. Furthermore, in unpublished data from our lab, mouse embryonic hippocampal gene transcription was compared for *Ptchd1* and Hh-signalling pathway genes *Shh*, *Smo*, and *Ptch1*. While the Hh pathway genes show decreasing transcription levels after E12 to birth, *Ptchd1* exhibits the opposite trajectory, with transcription increasing steadily from E12 to P2 (Supplementary Figure S1). Collectively, these support the notion that PTCHD1 operates in pathways other than Hh signalling.

The predicted PDZ-binding motif at the C-terminus of PTCHD1 is also present in SEC8 (also known as EXOC4), a component of the exocyst complex, where it facilitates interaction with the PDZ domains of the postsynaptic proteins PSD95 and SAP102 [45]. Correspondingly, Ung et al. sought to investigate if the PDZ-binding motif in Ptchd1 also mediates a similar interaction with both Psd95 and Sap102. Affinity purification experiments in synaptoneurosomal lysates from the adult mouse cortex confirmed that Ptchd1 interacts with both Psd95 and Sap102 in vitro and that this interaction is reliant on the PDZ-binding motif [33]. In order to identify additional proteins agnostically that may be putatively interacting with Ptchd1 in vitro, Tora et al. performed affinity purification in adult mouse whole brain lysates, followed by liquid chromatography-mass spectrometry. As bait, the authors used the final 43 amino acids of Ptchd1 and also a C-terminal variant in which they deleted the PDZ-binding motif (ΔPDZ). This approach identified numerous additional proteins that preferentially interacted with the wildtype C-terminal bait fragment, including novel components of the postsynaptic density (Dlg1-3, Magi1, Magi3, and Lin7), as well as components of the retromer complex (Snx27 and Vps26b). Furthermore, the additional retromer complex protein Vps35 was found to interact equally with both the wildtype and the ΔPDZ C-terminal bait fragments. Subsequent western blot analyses confirmed binding between the Ptchd1 C-terminus and both Psd95 and Vps35, with the latter interaction being independent of the PDZ-binding motif. Lastly, endogenous Ptchd1 was reported to be enriched in the postsynaptic density, further supporting its putative interaction with these proteins under physiological conditions [32].

Xie et al. employed a yeast two-hybrid screen to identify binding partners for the luminal loops of PTCHD1-binding proteins in vitro. Using bait fragments consisting of the first lumenal loop (F49-R270) and a chimera of both lumenal loops (F49-R270::Q521-S695), these authors found consistent interactions between PTCHD1 and the SNARE-associated protein (SNAPIN). This putative interaction was further corroborated by immunofluorescent studies in neuronally differentiated P19 cells, which demonstrated colocalization of exogenous PTCHD1 and SNAPIN in dendritic projections [37]. SNAPIN is involved in synaptic vesicle docking and fusion and is also a component of the biogenesis of lysosome-related organelles complex 1 (BLOC-1) complex [46], suggesting that PTCHD1 may play a role in the endosomal-lysosomal system.

### 2.6. PTCHD1-AS Function

As *PTCHD1-AS* does not encode for a protein, its contribution to the etiology of ASD and ID is unclear. Emerging evidence suggests that lncRNAs govern the expression of coding genes by altering chromatin status, influencing the initiation of transcription, as well as by affecting mRNA stability post-transcriptionally [47]. To investigate this, Ross et al. employed RNA fractionation and qRT-PCR to conclude that *PTCHD1-AS* was almost entirely localized to the nucleus, where it was also found to primarily associate with chromatin. In addition, given the proximity of the 5′ exons, the common bi-directional promoter, and their modest sequence homology, it seems plausible that *PTCHD1-AS* could be governing the expression of *PTCHD1* in *cis* to some extent. However, data from Ross et al. report that basal *PTCHD1* transcription was unchanged in male iPSC-derived cortical neurons from a patient with a 125 Kb microdeletion that encompassed exon 3 of *PTCHD1-AS (PTCHD1-AS*^Δ*3/Y*^). Next, in order to prematurely terminate transcription, these authors used CRISPR/Cas9 and homology-directed repair to replace exon 3 of *PTCHD1-AS* with two tandem polyadenylation sequences in iPSCs derived from an unaffected male *(PTCHD1-AS*^Δ*3-pA/Y*^) and subsequently observed a profound decrease in neuronal *PTCHD1* expression. These data suggest that, while *PTCHD1-AS* exon 3 may not necessarily be implicated in regulating *PTCHD1* expression, downstream portions of the antisense transcript could be involved. Curiously, transcripts bearing exons 5 and 6 of *PTCHD1-AS* were still detected in these CRISPR-edited cortical neurons, albeit at a reduced level, proposing the possibility of an additional TSS that is downstream of exon 3. To assess the global alterations in gene expression that are mediated by *PTCHD1-AS* in *trans*, Ross et al. performed microarray-based analyses on cortical neurons from iPSCs derived from the aforementioned male proband, as well as from an additional male ASD case with a 167 Kb microdeletion that eliminates the first two exons of *PTCHD1-AS2* and *PTCHD1-AS3*, as well as the first exon of *PTCHD1*. These investigations identified a paucity of abnormally expressed genes in cortical neurons derived from either proband, implying that *PTCHD1-AS* does not significantly affect global neuronal gene expression in *trans*. In addition, of the few dysregulated genes that were identified, none had any annotated neuronal function [35].

### 2.7. Ptchd1 Mutant Mouse Models for Cognitive and Metabolic Phenotypes

There is a considerable accumulation of evidence to suggest that *PTCHD1* is required for normal neurodevelopment; however, its mechanistic association with the etiology of ASD and ID remains poorly understood. To investigate this, numerous studies have used *Ptchd1* mutant (*Ptchd1^-/Y^*) mice in order to evaluate the effects of *Ptchd1* on behaviour, cognition, metabolism, gene expression, and both neuronal and synaptic structure and function. In order to disrupt *Ptchd1* in mice, multiple groups have independently generated a conditional allele by targeting exon 2 (*Ptchd1*^Δ*2/Y*^), which encodes three of the 12 transmembrane domains and a portion of one of two predicted sterol-sensing domains. Exon 2 consists of 661 nucleotides, and therefore, the resulting *Ptchd1*^Δ*2*^ transcript will have a premature truncation ahead of the final nine transmembrane domains, the sterol-sensing domain, and the PDZ-binding motif [31,32,33]. However, it should be noted that the removal of exon 2 still permits the generation of the shorter transcript encoding the 542 amino acid ORF and other more C-terminal ORFs (Figure 3A). Our own studies of brain tissue from the *Ptchd1^-/Y^* mice, courtesy of Guoping Feng, indicate that there is no loss of *Ptchd1* transcripts; however, while the *Ptchd1-a* transcript is lost, there is activation (~80-fold) of the shorter *Ptchd1-c* transcript (Vincent lab, *unpublished data*). Whether or not this transcript (or indeed the full-length transcript) is translated into protein has yet to be established.

**Figure 4:**
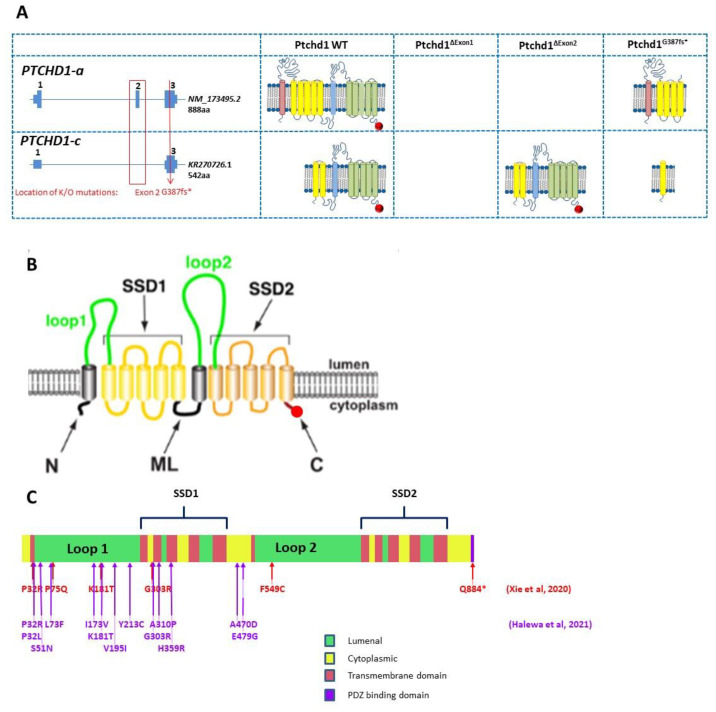
(**A**) Genomic organization of Ptchd1 isoforms a and c and predicted translation products resulting from the Murakami et al. [48] exon 1 knockout mice, Wells et al. [31] and Ung et al. [33] exon 2 knockout mice, and the CRISPR/cas9 knockout mouse generated by Frankland and Vincent [49]. The PDZ-binding motif is indicated by a red circle. N.B. Ptchd1-c transcription is activated (>80-fold) in the brains of the Wells et al. mice [31]. There are no predicted translation products for the exon 1 knockout [48], as the predicted promoter is also disrupted, and no additional promoters are predicted (genomatix.com, accessed on 2 August 2011). (**B**) Cartoon of PTCHD1 illustrating the predicted topological orientation of specific regions in the membrane; putative sterol-sensing domains (SSD) 1 and 2, luminal loops 1 and 2, medial loop (ML), N and C-termini, are shown. (**C**) Localization of missense variants studied in Halewa et al. [16] and Xie [37]. A more complete list of *PTCHD1* SNVs, including those identified in the MSSNG study, or reported by ClinVar, can be found on the www.PTCHD1-base.com (accessed on 1 February 2022) website.

In order to recapitulate clinical models where CNVs have encompassed just exon 1 or exon 3 of *PTCHD1* [5,6,9,12], exon-specific deletion mice have been generated. Murakami et al. generated mice with an exon 1 deletion (*Ptchd1*^Δ*1/Y*^) [48], while Ko et al. generated mice with an exon 3 deletion (*Ptchd1*^Δ*3/Y*^) [49]. Lastly, Roy et al. employed Cre-dependent SpCas9 adeno-associated viruses (AAVs) to knockdown *Ptchd1* (*Ptchd1^KD^*) in specific brain subregions in vivo [13]. The differences in the predicted effects of these knockout strategies on Ptchd1 protein manufacture are outlined in Figure 4.

A multitude of studies investigating the behavioural and neuromotor phenotype of male *Ptchd1^-/Y^* (i.e., Ptchd1^Δ*1/Y*^, Ptchd1^Δ*2/Y*^, and Ptchd1^Δ*3/Y*^) mice have identified numerous perturbations, many of which recapitulate the clinical symptoms of ASD and/or ADHD. *Ptchd1^-/Y^* mice demonstrate spontaneous hyperactivity, as inferred from an increase in the total distance travelled in a novel environment during an open field test (OFT) [48,49], and elevated locomotor activity [31,33].

The *Ptchd1*^Δ*1/Y*^ mice displayed ADHD-like behavior, exhibiting difficulty in habituating to new environments. Whereas control mice exhibit reduced exploration upon repeated exposure to an OFT, this reduction is attenuated in *Ptchd1*^Δ*1/Y*^ mice. Furthermore, the *Ptchd1*^Δ*1/Y*^ mice displayed heightened impulsivity, as determined by decreased jump latency in the cliff avoidance test [48]. Interestingly, treatment of the *Ptchd1*^Δ*1/Y*^ mice with the norepinephrine reuptake inhibitor Atomoxetine, which has been used to treat ADHD, was found to reduce, but not abolish, spontaneous hyperlocomotor activity in both the OFT and the cliff avoidance tests. Furthermore, Atomoxetine was also observed to normalize habituation of exploration in the OFT and reduce impulsivity in the *Ptchd1*^Δ*1/Y*^ mice [48].

The *Ptchd1*^Δ*2/Y*^ mice exhibited reduced anxiety, as evident from more time spent in the centre of the field during an OFT [33], although this phenotype was not replicated in *Ptchd1*^Δ*1/Y*^ mice [48]. Motor function defects were also reported in *Ptchd1*^Δ*2/Y*^ mice—Wells et al. report that *Ptchd1*^Δ*2/Y*^ mice exhibit altered gait parameters as well as signs of hypotonia, which was inferred from decreased grip strength, as assessed by either the hanging wire test [31] or an isometric dynamometer [33]. Ung et al. reported that *Ptchd1*^Δ*2/Y*^ mice presented with impaired motor coordination according to a rotarod test [33], although this was not detected by Wells et al. [31] Lastly, Wells et al. reported that *Ptchd1*^Δ*2/Y*^ mice exhibit a fragmented sleep pattern and hyper-aggressiveness, as determined by a longer attack duration and a shorter latency to attack during the resident intruder test, but the sensorimotor gating function remains unaffected (prepulse inhibition of the acoustic startle response [31]).

Cognitive assessments of male *Ptchd1*^Δ*1/Y*^ and *Ptchd1*^Δ*2/Y*^ mice reveal impairments related to learning and memory. Both *Ptchd1*^Δ*1/Y*^ and *Ptchd1*^Δ*2/Y*^ mice display diminished short-term working memory, which was inferred from fewer spontaneous alternations during the Y-maze test [33,48]. The Morris water maze test did not reveal deficits in spatial learning and memory in *Ptchd1*^Δ*2/Y*^ mice [31], although a targeted knockdown of *Ptchd1* in the anterodorsal (AD) thalamus suggested diminished spatial working memory, as inferred from fewer correct alternations during the T-maze test. Interestingly, this deficit was only present when the sample and choice trials were separated by a longer (60 s) interval, but a similar performance was observed when the inter-trial duration was short (10 s). Furthermore, these AD thalamus *Ptchd1^KD^* mice also demonstrated impaired long-term memory recall, which was evident from a significantly reduced freezing when tested 24 h after training in a contextual fear conditioning paradigm [13]. Notably, a reduced discrimination index on the novel object recognition test (NORT) provides evidence of weakened recognition memory in both *Ptchd1*^Δ*1/Y*^ and *Ptchd1*^Δ*2/Y*^ mice [32,33,48]. Interestingly, Atomoxetine treatment rescued this impairment in recognition memory, as evident by less interaction time with the familiar object on the NORT in *Ptchd1*^Δ*1/Y*^ mice [48]. Moreover, *Ptchd1*^Δ*2/Y*^ mice demonstrated additional learning impairments, as indicated by a diminished latency to cross during the inhibitory avoidance task [31] and decreased freezing in contextual and cued conditioning paradigms [31,33] (similar to the AD thalamus *Ptchd1^KD^* mice).

Interestingly, *Ptchd1* disruption appears to be associated with deficits in attentional engagement and sensory filtering. These deficits presumably contribute to learning and memory impairments, as *Ptchd1*^Δ*2/Y*^ mice are impaired in a cognitive task in the presence of a visual distractor, suggesting sensory-related distractibility in these mice [31]. Similarly, *Ptchd1*^Δ*2/Y*^ mice demonstrated a markedly poorer ability to discriminate between different auditory stimuli when provided with high levels of background noise, both in the presence and absence of preceding visual cues [50].

The studies by Wells et al. have implicated the TRN as a key brain region in several of the behavioural and cognitive abnormalities observed in *Ptchd1*^Δ*2/Y*^ mice. When the deletion of *Ptchd1* exon 2 was principally confined to the TRN using *Somatostatin*-driven *Cre* mice (*Som-Cre^+^:Ptchd1^fl/Y^*), hyperactivity, problems with attentional engagement, and fragmented sleep all persisted, indicating that *Ptchd1* expression in the TRN is critical for these functions. Notably, another type of learning and memory (i.e., inhibitory avoidance task) was found to improve, and both hyper-aggression and hypotonia were no longer detected in these *Som-Cre^+^:Ptchd1^fl/Y^* mice, suggesting that *Ptchd1* expression elsewhere in the brain influences these traits [31].

Murakami et al. investigated the possible relationship between *Ptchd1*-mediated metabolic dysregulation and ADHD-like behavioral phenotypes. These authors specifically focused on the effects of *Ptchd1* on the kynurenine pathway (KP), which metabolizes tryptophan to generate nicotinamide adenine dinucleotide (NAD^+^), a coenzyme that is critical for redox reactions in both metabolism and energy production. In 11-week-old *Ptchd1*^Δ*1/Y*^ mice, these authors detected increased serum concentrations of kynurenine, as well as an increased presumptive activity of the enzyme indoleamine-2,3-oxygenase1 (Ido1), the first and rate-limiting KP enzyme. Notably, atomoxetine, a selective norepinephrine reuptake inhibitor, typically used as a treatment for ADHD, ameliorated both the heightened activity of Ido1 and the elevated concentration of kynurenine in the serum of the *Ptchd1*^Δ*1/Y*^ mice. In addition to the serum, elevated levels of the KP metabolites 3-hydroxykynurenine (3-HK), anthranilic acid (AA), and 3-hydroxyanthranilic acid (3-HAA) were all detected in the *Ptchd1*^Δ*1/Y*^ frontal cortex. Atomoxetine administraton also reduced the levels of AA in the *Ptchd1*^Δ*1/Y*^ frontal cortex, although the concentrations of both 3-HK and 3-HAA remained abnormally high. Interestingly, atomoxetine was observed to significantly raise the concentration of an additional KP metabolite, kynurenic acid (KYNA), in the *Ptchd1*^Δ*1/Y*^ frontal cortex. Collectively, these data suggest an association between the dysregulation of tryptophan metabolism and *PTCHD1*-related ASD and/or ID; however, this association has yet to be confirmed either in *PTCHD1*-deficient patients or in other *Ptchd1* mouse models [20].

Unexpectedly, given the *PTCHD1* involvement in ASD, no social abnormalities were apparent in either *Ptchd1*^Δ*1/Y*^ or *Ptchd1*^Δ*2/Y*^ mice as inferred from the three-chambered social interaction assay [31,48] and the social recognition test [33]. Despite this, evidence of possible stereotypic behaviours was reported in *Ptchd1*^Δ*2/Y*^ mice, including more frequent rearing and increased locomotor activity in the back portion of the cage during the active part of the circadian cycle [33], although repetitive grooming was not observed [31].

However, a preliminary study by Ko et al. suggests potential PTCHD1/Ptchd1 involvement in both ASD and ID, with respect to both social abnormalities and learning deficits in mice [49]. Based on loss CNV and nonsense mutation cases reported in PTCHD1-related ASD patients [6], they generated *Ptchd1* exon 3 truncating mutation mice using CRISPR-Cas9 technology (*Ptchd1*^Δ*3/Y*^). *Ptchd1*^Δ*3/Y*^ mice exhibited significant reductions in both *Ptchd1* full-length (i.e., the transcript derived from exons 1 to 3) and shorter transcripts (i.e., the transcript derived from exons 1 and 3), and social interaction and communication behaviors were abnormal in these mice, as inferred from the reduced male-female interaction time in the three-chambered social interaction assay, the reduced emission of ultrasonic vocalization during the social interaction, and the reduced sniffing behavior in the social odor cue reactivity task, respectively. Furthermore, *Ptchd1*^Δ*3/Y*^ mice also exhibit impaired learning and memory in contextual fear conditioning [49]. Given that *Ptchd1*^Δ*2/Y*^ mice display significant enrichment of the shorter alternatively spliced form of the *Ptchd1* transcript (*Ptchd1-c*) and that *Ptchd1*^Δ*3/Y*^ mice lack expression of both full-length (*Ptchd1-a*) and shorter form (*Ptchd1-c*) transcripts, it seems plausible that the more complete phenotypic picture in the *Ptchd1*^Δ*3/Y*^ mice (i.e., including social deficits) relates to the loss of both major alternative transcripts (*Ptchd1-a* and *-c*). In other words, it is plausible that the increased expression of *Ptchd1-c* in the *Ptchd1*^Δ*2/Y*^ mice rescues the social deficit phenotypes.

### 2.8. Neurodevelopmental Implications of PTCHD1 and PTCHD1-AS

Extensive efforts have sought to determine the neurophysiological mechanisms of *Ptchd1*-mediated phenotypic abnormalities. Pioneering work by Wells et al. in 2016 implicated the TRN as being a critical region in the brain that underlies many of the behavioural and cognitive abnormalities displayed in *Ptchd1*^Δ*2/Y*^ mice. In TRN neurons of *Ptchd1*^Δ*2/Y*^ mice, whole-cell patch clamp recordings reveal a decrease in repetitive bursting. In addition, *Ptchd1*^Δ*2/Y*^ TRN neurons exhibited a lower rate of burst firing during sleep, which translated into an overall reduction in sleep spindles and a fragmented sleep pattern. Subsequent analyses indicate that this lowered burst rate may be a consequence of reduced ion currents traveling through small conductance Ca^2+^-activated K^+^ (SK) channels. Furthermore, a two-fold lower basal concentration of intracellular Ca^2+^ was observed, which may also contribute to the deficits observed in SK channels [31]. Moreover, the insufficient hyperpolarization that results from these reduced K^+^ currents likely impairs the voltage-gated recruitment of T-type Ca^2+^ channels [51], which may affect the excitability of the TRN. The TRN is a GABAergic group of neurons that provides the principal source of inhibition to the thalamic relay nuclei, and therefore, diminished TRN function could contribute to attenuated thalamic inhibition [52]. One possible implication of reduced thalamic inhibition is an inability to suppress unwanted sensory stimuli [53], which may help to explain the heightened distractibility in *Ptchd1*^Δ*2/Y*^ mice [31]. Interestingly, pharmacological augmentation of SK currents in *Ptchd1*^Δ*2/Y*^ mice using the agonist 1-ethyl-2-benzimidazolinone (1-EBIO) significantly increased sensory-evoked thalamic inhibition, which mitigated their heightened distractibility and improved cognitive performance in the presence of visual distractors [31].

To elaborate on the apparent thalamic dysregulation and distractibility in *Ptchd1*^Δ*2/Y*^ mice, Nakajima et al. further probed the affiliation between *Ptchd1* disruption and thalamic-mediated noise hypersensitivity by examining the auditory subnetwork of the TRN (audTRN). These analyses reveal that the sound-evoked firing rates of audTRN neurons were diminished in *Ptchd1*^Δ*2/Y*^ mice. Interestingly, the impairment in the ability of *Ptchd1*^Δ*2/Y*^ mice to discriminate between auditory stimuli in the presence of high levels of background noise was fully rescued by 1-EBIO supplementation, but only when anticipatory visual cues were not provided before the auditory stimulus. Unexpectedly, when prior visual cues were given, 1-EBIO only elicited a marginal improvement in the ability of *Ptchd1*^Δ*2/Y*^ mice to filter out unwanted background noise. This finding suggests a deficit of executive control over sensory filtering in *Ptchd1*^Δ*2/Y*^ mice, which may be mediated by the prefrontal cortex (PFC). Remarkably, a synergistic approach that used the cognitive enhancer modafinil in combination with 1-EBIO was observed to fully restore both the PFC and the audTRN function, as well as to rescue the attenuated discrimination performance in the *Ptchd1*^Δ*2/Y*^ mice when visual cues were provided [50].

Roy et al. subsequently used ex vivo electrophysiology to examine neuronal properties in the *Ptchd1^KD^* AD thalamus and identified a reduction in action potential half-width and a corresponding increase in firing frequency [13]. These authors also report that a contextual fear-conditioning paradigm did not elicit synaptic strengthening in the *Ptchd1^KD^* AD thalamus, as evidenced by a stagnant AMPA-to-NMDA (A/N) ratio in the AD thalamus-retrosplenial cortex (RSC) circuit. This diminished synaptic strengthening appears to be a consequence of the hyperexcitability of neurons in the *Ptchd1^KD^* AD thalamus. Synaptic strengthening during learning is dependent upon a stimulus-induced increase in the excitability of AD neurons, and the abnormally high basal excitability of *Ptchd1^KD^* AD thalamic neurons may occlude learning-induced changes. They showed that *Ptchd1^KD^* impairs the learning-dependent A/N ration and the *c-fos* expression, but pharmacological inhibition of AD thalamic- > RSC circuits in *Ptchd1^KD^* mice rescued the A/N ratio and *c-fos* levels in the RSC but not in the AD thalamus. Next, to determine the molecular mechanism for this apparent hyperexcitability in *Ptchd1^KD^* AD thalamic neurons, Roy et al. used FISH and elucidated that the expression of two transcripts, *Cacna1a* and *Cacna1b*, that encode for voltage-gated P-type calcium channel subunits alpha-1A (which contain the Ca^2+^ pore of Ca_v_2.1 channels) and alpha-1B (Ca_v_2.2 channels), respectively, were both markedly lower in these neurons. Subsequent ex vivo electrophysiology revealed reduced Ca_v_2.1 and Ca_v_2.2 current amplitudes in *Ptchd1^KD^* AD thalamic neurons [13].

In addition to the thalamus, considerable research has been devoted to another brain subregion, the hippocampus, in an effort to comprehend the memory impairments that have been reported in *Ptchd1^-/Y^* (exon 2 deletion) mice. In the hippocampus, Tora et al. were unable to detect differences in the density or morphology of glutamatergic dendritic spines in dentate granule cells in P21 and P60 *Ptchd1*^Δ*2/Y*^ mice [32]. In contrast, Ung et al. used transmission electron microscopy to show a decrease in the density of excitatory synapses, as well as ultrastructural attenuations in both postsynaptic density thickness and synaptic cleft width in the hippocampal synapses of *Ptchd1*^Δ*2/Y*^ mice that were 13–15 weeks old. Interestingly, these authors also report a significant accumulation of neurotransmitter vesicles in *Ptchd1*^Δ*2/Y*^ synaptic boutons. Lastly, Ung et al. evaluated the morphology of GFP-labelled *Ptchd1*^Δ*2/Y*^ primary hippocampal neuronal cells that were differentiated for 18 days ex vivo and identified decreased dendritic length and branching arborisation [33]. Functionally, whole-cell voltage clamping of P21–P24 hippocampal slices revealed a reduction in the excitation-to-inhibition ratio of *Ptchd1*^Δ*2/Y*^ dentate granule cells, as well as a basal increase in the frequency of both spontaneous excitatory (sEPSC) and inhibitory (sIPSC) potentials [32]. Despite this apparent increase in basal currents in dentate granule cells, a substantial decrease in the frequency of evoked miniature excitatory postsynaptic currents (mEPSCs) was observed in the surrounding *Ptchd1*^Δ*2/Y*^ CA1 pyramidal neurons. Subsequently, the paired-pulse ratio indicates that *Ptchd1*^Δ*2/Y*^ Schaffer collateral axons, the primary excitatory input onto CA1 pyramidal cells, have a higher probability of vesicular release, which may mitigate the reduced frequency of mEPSCs [33].

To evaluate the effects of *Ptchd1* exon 2 deletion on the hippocampal transcriptome, Ung et al. performed RNA sequencing on hippocampal samples from P30 *Ptchd1*^Δ*2/Y*^ mice and identified a large number of aberrantly expressed genes [33]. These authors further used published single-cell transcriptomics data from the P21–P30 mouse hippocampus [54] in order to perform gene set enrichment analyses to identify specific hippocampus cell subtypes that may be particularly affected in the *Ptchd1*^Δ*2/Y*^ mice. This bioinformatic approach revealed that upregulated genes in the *Ptchd1*^Δ*2/Y*^ hippocampus were significantly enriched in the markers of neuronal genes, in particular pyramidal neurons and interneurons. Interestingly, downregulated genes in the *Ptchd1*^Δ*2/Y*^ hippocampus were significantly enriched in the markers of glial cells, specifically astrocytes and oligodendrocytes, as well as endothelial cells. A subsequent gene ontology enrichment analysis demonstrated that the array of upregulated neuronal genes in the *Ptchd1*^Δ*2/Y*^ hippocampus was significantly enriched in genes encoding synaptic proteins. Specifically, abnormal upregulation was observed for approximately 20% of genes that encode presynaptic proteins, such as *Syt1*, *Bsn*, *Vamp3*, and *Syn1-3*. Similarly, the expression of almost 25% of the genes encoding the postsynaptic proteins was likewise found to be augmented, including *Dlg4* (which encodes for Psd95), as well as *Camk2a*, *Syngap1*, and *Shank1-3*. In addition to synaptic protein-coding genes, the authors also report an upregulation of genes encoding proteins involved in different aspects of nervous system development, such as axonogenesis and dendritogenesis. Lastly, increases in the expression of genes encoding the neuronal activity-dependent transcription factors *Npas4* and *Egr1* were also discovered in the *Ptchd1*^Δ*2/Y*^ hippocampus. Taken in aggregate, these data suggest that the absence of *Ptchd1* in hippocampal neurons may lead to the dysregulation of neuronal and synaptic structure and function [33].

In order to examine the neurophysiological function of *PTCHD1* and *PTCHD1-AS* in a human context, Ross et al. sought to characterize the neuronal properties of iPSCs reprogrammed from male ASD probands with previously characterized microdeletions in Xp22.11 [11]. No apparent alteration in the dendritic morphology was observed in cortical neurons from iPSCs derived from either of the two aforementioned male ASD probands (with 125 Kb (*PTCHD1-AS* exon 3) and 167 Kb (*PTCHD1-AS* exons plus exon 1 of *PTCHD1*) loss CNVs). Interestingly, *PTCHD1-AS*^Δ*3/Y*^ cortical neurons appeared to display an increased density of excitatory synapses within dendrites. Furthermore, in vitro electrophysiological analyses indicated that CRISPR/cas9-edited *PTCHD1-AS*^Δ*3-pA/Y*^ cortical neurons (in which *PTCHD1-AS* exon 3 has been replaced with tandem polyadenylation sites), as well as those derived from both the 125 Kb and 167 Kb loss CNV probands, all exhibited marked attenuations in AMPAR-mediated mEPSC frequency, with an additional reduction in mEPSC amplitude observed in the CRISPR-edited cortical neurons. Lastly, cortical neurons from both probands also demonstrated a reduction in NMDA-evoked current amplitude [35].

## 3. Summary and Future Research Directions

This review provides an overview and timeline of the clinical genomic investigations that have identified *PTCHD1* and *PTCHD1-AS* as risk factor genes for the etiology of ASD and ID. In numerous male ASD and ID cases, inherited and de novo microdeletions in Xp22.11 affecting all or part of *PTCHD1*, *PTCHD1-AS*, or both have been reported, as have point mutations and indels in the *PTCHD1* coding sequence that have generated missense, nonsense, or truncating variants. Loss-of-function mutations affecting *PTCHD1* appear to segregate with disease in the standard X-linked (recessive) mode of inheritance, suggesting a strong causative relationship with the corresponding phenotypes. In contrast, loss CNVs affecting *PTCHD1-AS* without disrupting *PTCHD1* may show full, incomplete, or no segregation and are present in control populations, making the association between *PTCHD1-AS* and disease either complicated or possibly tenuous. Clinically, we have also described the dysmorphic, cognitive, behavioural, and neurological features of several patients with a variety of mutations affecting *PTCHD1*.

*PTCHD1* is widely transcribed throughout the human brain, with the highest level of expression being observed in the cerebellum and pituitary gland. Similarly, *PTCHD1-AS1* and *PTCHD1-AS2* transcripts have also been detected in several brain regions. In mice, *Ptchd1* is transcribed throughout the developing brain during embryogenesis. At birth, *Ptchd1* expression is most pronounced in the TRN but by adulthood becomes primarily abundant in the granule cells of both the dentate gyrus and the cerebellum. Notably, the expression of *PTCHD1*, but not *PTCHD1-AS*, has been found to be enhanced by neuronal depolarization in vitro, inferring a possible activity-dependent function.

PTCHD1 contains 12 predicted transmembrane domains and exogenously expressed PTCHD1 has been observed to be directed to the plasma membrane within dendritic spines in neuronal cultures. Furthermore, this localization is apparently reliant on a portion of the cytoplasmic C-terminal tail. Functionally, despite amino acid sequence homology with PTCH1 and PTCH2, PTCHD1 does not appear to be involved in negatively regulating the Shh signalling pathway. The C-terminal PDZ-binding motif of Ptchd1 can mediate interaction with numerous components of both the retromer complex and the postsynaptic density, suggesting a potential involvement in endosomal protein sorting within dendritic spines.

Studies involving *Ptchd1^-/Y^* mice have revealed that these mice display behavioural, neurological, and cognitive abnormalities, many of which recapitulate the clinical symptoms of ASD and/or ADHD. These aberrant traits include hyperactivity, hyper-aggression, motor defects, hypotonia, learning, and memory impairments, as well as deficits in attentional engagement and auditory sensory filtering; social deficits, however, have only been reported for the *Ptchd1 Ptchd1*^Δ*3/Y*^ exon 3 knockout mice [49].

Neurophysiological dysregulation in the brain may underlie the perturbations in sensory filtering in *Ptchd1*^Δ*2/Y*^ mice. In the absence of *Ptchd1*, reduced K^+^ efflux through SK channels leads to impaired hyperpolarization, which affects the activity of T-type Ca^2+^ channels and subsequently reduces TRN-mediated thalamic inhibition. This reduced thalamic inhibition impairs the ability of *Ptchd1*^Δ*2/Y*^ mice to suppress unwanted sensory stimuli, thus leading to increased distractibility. Furthermore, both the automatic and the executive mechanisms of auditory filtering are diminished in *Ptchd1*^Δ*2/Y*^ mice, with the former being regulated by the audTRN and the latter being mediated by the PFC.

The AD thalamus and hippocampus may be implicated in memory deficits in the absence of *Ptchd1*. Local *Ptchd1* knockdown in the AD thalamus leads to neuronal hyperexcitability, which was facilitated by dysfunction of Ca_v_2.1 and Ca_v_2.2. Likewise, this hyperexcitability also attenuated learning-induced synaptic plasticity in these mice, leading to diminished long-term memory. Similarly, *Ptchd1*^Δ*2/Y*^ hippocampal neurons displayed altered dendritic and synaptic morphology. Moreover, the hippocampal transcriptome of *Ptchd1*^Δ*2/Y*^ mice exhibited upregulation in genes encoding for both pre- and postsynaptic proteins. Unsurprisingly, these structural and transcriptomic defects in the hippocampus were functionally accompanied by a reduction in neuronal excitability.

While recent studies into the neurodevelopmental contributions of *PTCHD1* and *PTCHD1-AS* have increased dramatically over the past decade, there is still much to learn about the molecular interactions and pathways involved. Studies involving *Ptchd1*^Δ*2/Y*^ mice have clarified the role of *Ptchd1* in the neurophysiological etiology of cognitive and attentional impairments, which are both clinical characteristics of ASD and ID. However, analyses for the *Ptchd1*^Δ*2/Y*^ mice have failed to replicate the analogous social deficits that have been reported in ASD cases involving *PTCHD1* mutations, whereas the preliminary evidence for *Ptchd1*^Δ*3/Y*^ mice indicates a clear social deficit. It is therefore crucial to understand the differences in the *Ptchd1* gene product at the mRNA and protein level for the different mouse models. Further research is also required to identify the mechanisms by which these genes influence the broad spectrum of social abnormalities that underlie ASD, with a special focus on the development of pharmacological and cognitive interventions to mitigate neurophysiological dysfunction. In addition, many fundamental questions persist regarding the specific biochemical and molecular functions of PTCHD1 within neurons. Specific domains within PTCHD1, for instance the predicted sterol-sensing domains, the two major luminal loops, and the N- and C-termini (Figure 4B), still have no clearly identified role.

In summary, disruption of *PTCHD1* is strongly associated with the etiology of both ASD and ID in humans and in mice with behavioural and learning deficits strongly reminiscent of ASD and ID, and thus, it is likely to be important for normal neurodevelopment. Further molecular and cellular and neuroanatomical characterization is essential before tackling strategies for pharmacological interventions to ameliorate these conditions.

## Data Availability

CNV data from the PGC schizophrenia study (Marshall et al, 2017) were made available upon request to www.tcag.ca (accessed on 1 December 2021). gnomAD non-neuro control CNV data were accessed through https://gnomad.broadinstitute.org (accessed on 1 December 2021). CNV data from 1000 Genome Project phase 3 were accessed from https://www.internationalgenome.org/data/(accessed on 1 December 2021). MSSNG CNV data was accessed by agreement with Autism Speaks- downloaded data are available on our PTCHD1-base.com website, also by request to the authors. Other data were access directly from cited publications, or from ClinVar (https://www.ncbi.nlm.nih.gov/clinvar/, accessed on 1 August 2021), or from DECIPHER (https://www.deciphergenomics.org/, accessed on 1 August 2021). Transcriptional data can be accessed through https://genome.ucsc.edu (accessed on 1 February 2022).

## Supplementary Materials

The following supporting information can be downloaded at: https://www.mdpi.com/article/10.3390/genes13030527/s1, Figure S1: RT-qPCR of mouse embryonic brain cDNA at different developmental time-points (E12 to P2) for *Ptchd1* as well as Shh-signalling pathway genes, *Shh*, *Smo*, and *Ptch1*. Table S1: Full list of cited *PTCHD1*/*PTCHD1-AS* variants.
