# Supplementary material for: PTCHD1: Identification and Neurodevelopmental Contributions of an Autism Spectrum Disorder and Intellectual Disability Susceptibility Gene
Source: Genes (Basel). 2022 Mar 17;13(3):527. doi: 10.3390/genes13030527 (PMC8953913; doi:10.3390/genes13030527)
Supplement: Supplementary file 1 [file genes-13-00527-s001.zip › Supplementary Information_21Feb2022.pdf]

Supplementary Information

Supplementary Figure S1: RT-qPCR of mouse embryonic brain cDNA at different developmental time-points (E12 to P2) for Ptchd1 as well al Shh-signalling pathway genes, Shh, Smo, and Ptch1. Expression is shown as mean fold-change (+/- standard error of the mean), with expression at E12 set at 1.

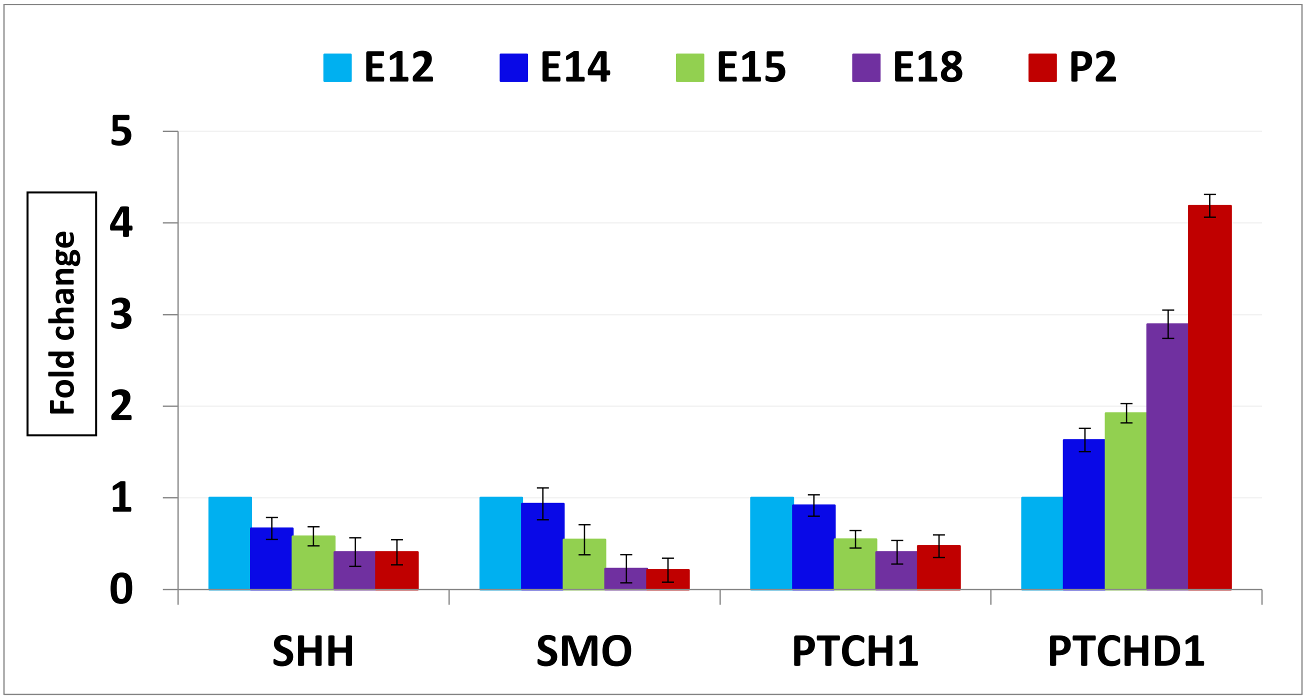

In order to characterize *Ptchd1* expression in WT mice, relative to Shh signaling genes *Ptch1*/*Shh*/*Smo*, offspring from CD-1 mice were used. The mice were mated and checked for sperm plugs. Tissue was collected (N=4 embryos/pups) at gestational days 12, 14, 15, 18 and postnatal day 2. RNA was extracted from whole brains using the Nucleospin RNAII kit (Macherey-Nagel Inc.). RNA concentration and purity were determined using the ratio of 260/280 nm and 260/230 nm, respectively, as determined by a Nanodrop^{TM} 1000 (Thermo Fisher Scientific). Only RNA with a 260/280 nm ratio of >1.90 was used in our analysis. cDNA was synthesized through reverse transcription of 1 µg of RNA using Superscript III^{TM} Reverse Transcriptase (Invitrogen, Carlsbad, CA) and random hexamers according to the manufacturer's guidelines. PCR was performed using 384-well optical plate, using 2 µl of cDNA with a final reaction volume of 16 µl run on a ViiA™7 Real Time PCR System (Life Technologies, Thermo Fisher Scientific). Universal SYBR Green_ PCR conditions were used (95 °C for 150 s, and 40 cycles at 95 °C for 4 s, and 60 °C for 20 s). For each gene analyzed all samples were run on a single plate to avoid inter-plate variability, and in quadruplicate. Furthermore, each plate contained H_2O, RT-minus and RNA-minus negative controls. The C_t for all reactions was

calculated automatically by the Life Technologies ViiA™7 software. Gene expression analysis was calculated using the $2^{-\Delta\Delta Ct}$ method (Litvak and Schmittgen, 2001).

Primers used:

Ptchd1 F: ACATCACACGGACCTGATCTTA

Ptchd1 R: CTATGTCATCCACAATGCAAGTC

Smo F: CTTATTGTGGGAGGCTACTTCCT

Smo R: TGGTCTCGTTGATCTTGCTG

Ptch1 F: TGGTTGTGGGTCTCCTCATATT

Ptch1 R: AATTCTCGACTCACTCGTCCAC

Actin F: CGTGCGTGACATCAAAGA

Actin R: TGCCACAGGATTCCATAC

Shh F: GATGACTCAGAGGTGCAAAGAC

Shh R: ACTGCTCGACCCTCATAGTGTA

References:

Livak KJ, Schmittgen TD. *Methods.* Vol. 25. San Diego, CA: 2001. Analysis of relative gene expression data using real-time quantitative PCR and the 2(-Delta Delta C(T)) Method; pp. 402–408
